# Clinical Decision Support and New Regulatory Frameworks for Medical Devices: Are We Ready for It? - A Viewpoint Paper

**DOI:** 10.34172/ijhpm.2021.144

**Published:** 2021-10-18

**Authors:** Sven Van Laere, Katoo M. Muylle, Pieter Cornu

**Affiliations:** ^1^Research Group of Biostatistics and Medical Informatics (BISI), Department of Public Health (GEWE), Vrije Universiteit Brussel, Brussel, Belgium.; ^2^Research Group Clinical Pharmacology and Clinical Pharmacy (KFAR), Centre for Pharmaceutical Research (CePhar), Vrije Universiteit Brussel, Brussel, Belgium.

 Use of Software as a Medical Device (SaMD) is continuously increasing. SaMD is defined by the International Medical Device Regulators Forum (IMDRF) as software intended to be used for medical purposes without being part of a hardware medical device.^1^ SaMD is software that can perform complex clinical tasks such as diagnosing medical conditions, suggesting treatments and informing clinical management.^1^

 In light of the rapidly changing digital health landscape, policy makers have acknowledged the need for an updated regulatory framework to ensure and promote safe innovation. In the United States, such a regulatory response was given in the 21st Century Cures Act, which was enacted in December 2016.^2^ In the European Union (EU), the Medical Device Regulation (MDR) followed in May 2017.^3^ This Regulation was due to be enforceable in all member states as of May 2020, but this was postponed with a year because of the coronavirus disease 2019 (COVID-19) pandemic.^4^ Both laws aim to set high quality and safety standards for medical devices and improved transparency and traceability.^2,3^

 In this paper, we will focus on the impact of these legislations on clinical decision support (CDS) software as a specific type of SaMD. We define CDS as any software system that integrates personal patient data with external sources of medical knowledge to assist healthcare professionals in their decision-making process.^5^ Such an external source may be well-established information such as clinical guidelines, but it can also be information inferred by an algorithm using machine learning or artificial intelligence techniques. CDS can be either stand-alone or integrated in another medical device.^5^

## 21st Century Cures Act: Device CDS and Non-device CDS

 In the United States, controversy was raised around the implications of the Cures Act on CDS.^6,7^ The first major problem was that it was initially not clear which types of CDS would be regulated under the Cures Act and which ones would be exempted. Secondly, the new requirements were not always proportionate to the risk a particular CDS function might pose.

 The Food and Drug Administration (FDA) responded with the publication of a guidance on CDS software on September 27, 2019.^8^ In this document the FDA clarified their position on regulating different kinds of CDS software functions using a risk-based approach as suggested by the IMDRF Framework.^8,9^ In the guidance document, CDS is categorized into ‘Device CDS’ and ‘Non-device CDS’. Non-Device CDS is CDS that is exempted from the definition of medical device because it meets all of the following four criteria^8^:

1. Not intended to acquire, process, or analyze a medical image or a signal from an in vitro diagnostic device or a pattern or signal from a signal acquisition system;
2. Intended for the purpose of displaying, analyzing, or printing medical information about a patient or other medical information (such as peer-reviewed clinical studies and clinical practice guidelines);
3. Intended for the purpose of supporting or providing recommendations to a healthcare professional about prevention, diagnosis, or treatment of a disease or condition; and
4. Intended for the purpose of enabling such healthcare professional to independently review the basis for such recommendations that such software presents so that it is not the intent that such healthcare professional rely primarily on any of such recommendations to make a clinical diagnosis or treatment decision regarding an individual patient.

An example of a Non-Device CDS that the FDA considers to be exempted from regulation is software that identifies drug-drug interactions based on the recommendation of reliable medical sources.^8^ CDS for drug-drug interaction screening is often knowledge-based, comparing information from one or more validated drug-drug interaction databases to the medication a patient is currently taking and newly prescribed medication.

 Unfortunately, current CDS for drug-drug interaction screening is often too sensitive leading to a high alert burden with alert fatigue and high override rates.^10,11^ Improving the specificity of CDS for drug-drug interaction screening is an essential part of ongoing efforts to improve CDS’s effectiveness.^10,12^ Algorithms may rely on traditional statistical regression models or on more advanced machine learning or artificial intelligence techniques. For both, it is not clear whether the healthcare professional is truly capable of independently reviewing the basis of the recommendation, the key criterion that decides whether the CDS function is considered Device CDS or Non-device CDS. The FDA states that the manufacturer should describe the algorithm’s underlying data and include plain language descriptions of the logic or rationale used to produce a certain recommendation. When complex algorithms are used to determine the risk level of a drug-drug interaction, this would mean that the specific elements used (eg, lab values and comorbidities), should be displayed on the alert screen. When the logic of the algorithm is also explained and available to the healthcare professional (directly or on demand), the fourth criterion is fulfilled, and thus the software is exempted from the Cures Act. As Evans and Ossorio pointed out, the software manufacturer only needs to *intend* and does not need to *succeed* for its software to be transparent and explainable in its recommendations.^13^ It seems that the fourth criterion should thus be interpreted as whether the healthcare practitioner can theoretically understand or evaluate the basis for a certain recommendation.

 The FDA still has to further clarify how they will assess whether a certain CDS software is explained or not. This highly influences whether the manufacturer has to comply with FDA regulation or is exempted. In case the FDA would only exempt the simplest CDS software, the opposite effect of the intention of the Cures Act may be achieved, namely significant delayed access to new innovations and advances for patients.

## 21st Century Cures Act: Risk Classification

 Acknowledging the balance between timely patient access and safety, FDA adopted the risk-based classification of SaMD as proposed by IMDRF.^8,9^ This classification is based on two main factors: (*a*) the significance of the information provided by a SaMD to a certain type of healthcare decision, and (*b*) the state of the patient’s healthcare situation or condition, resulting in four risk categories. Category IV is considered the highest risk category, whereas SaMD with the lowest risk are classified as category I (Table).

**Table:** Risk Profiles as Defined in the 21st Century Cures Act for Software as Medical Device Ranging From Low Risk (Class I) to High Risk (Class IV)

**State of Healthcare Situation or Condition**	**Significance of Information Provided by SaMD to Healthcare Decision**		
	**Treat or Diagnose**	**Drive Clinical Management**	**Inform Clinical Management**
Critical	IV	III	II
Serious	III	II	I
Non-serious	II	I	I

Abbreviation: SaMD, software as medical device. Source: US Food & Drug Administration.^8^

 The guidance specifies that SaMD functions that drive clinical management or that treat or diagnose are *not* considered CDS as defined in the Cures Act, because criterion (3) is not met. Hence, CDS functions always belong to category I or II (Figure, section A). CDS functions that inform clinical management for *non-serious conditions* with patients or caregivers as intended users who can independently review the basis of the recommendations, are considered to be class I and low risk CDS functions. An example of this type of CDS is software that provides patients or caregivers a prioritized list of over-the-counter medications matching with their notified symptoms for a non-serious condition like common cold. Likewise, CDS functions for a non-serious condition intended for healthcare professionals that do not meet criterion (4), because the recommendations are not designed to be independently reviewed, are considered low risk. An example of such a CDS function would be an algorithm that uses patient-specific data such as blood test results and medication information to alert healthcare practitioners of cholesterol management issues. For this type of Device CDS functions, FDA does not intend to enforce compliance with the Cures Act (Figure, section A). Instead, FDA will focus its regulatory oversight on higher risk Device CDS functions informing clinical management for *serious* and *critical healthcare conditions*, like for example an unexplained algorithm that identifies hospitalized patients at increased risk of postoperative cardiovascular events.

**Figure:**
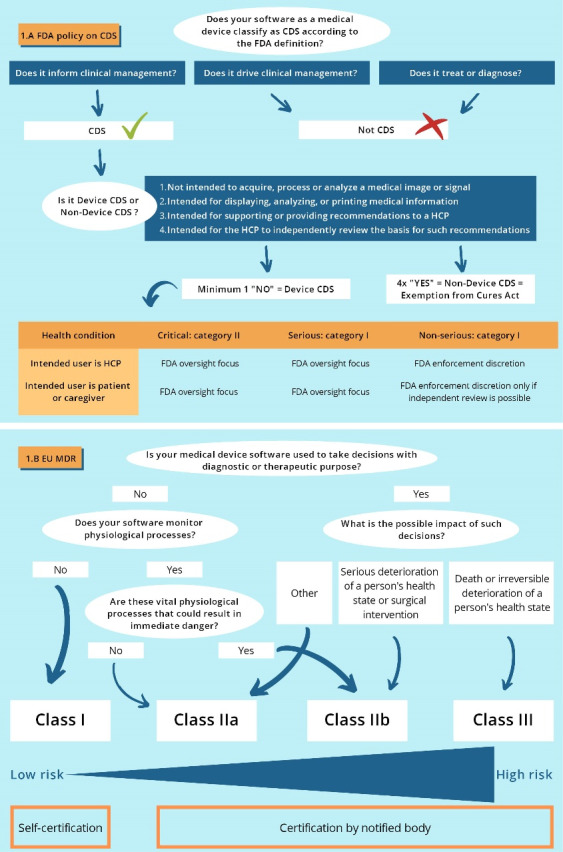
Infographic on Food and Drug Administration Policy on Clinical Decision Support According to the Food and Drug Administration Guidance Specifically for Clinical Decision Support^8^ (**A**) and on the Classification of Medical Device Software According to the European Union Medical Device Regulation (**B**). Abbreviations: CDS, clinical decision support; FDA, Food and Drug Administration; HCP, healthcare professional; MDR, Medical Device Regulation; EU, European Union. “FDA Enforcement Discretion” indicates that the FDA does not intend to enforce compliance, and “FDA oversight focus” indicates that the focus is on regulating CDS software by the FDA. In the United States, CDS functions belong to category I or II depending on the health condition and type of intended user. In the EU, medical device software with the lowest risk is classified as class I device, whereas class III represents the highest risk category. Only class I devices can be self-certified, other devices must be certified by a notified body.

## Europe’s Medical Device Regulation

 In Europe a similar medical device law, the MDR, was implemented in May 2021. Instead of the term SaMD, the term medical device software is used. According to the definition of medical devices any software with the purpose of: (1) diagnosis, prevention, monitoring, prediction, prognosis, treatment or alleviation of disease; (2) diagnosis, monitoring, treatment, alleviation of, or compensation for, an injury or disability; (3) investigation, replacement or modification of the anatomy or of a physiological or pathological process or state; and (4) providing information by means of in vitro examination of specimens derived from the human body, including organ, blood and tissue donations, falls under the new legislation.^3^ Under the MDR, software vendors should certify their products via notified bodies.^3,14^ These notified bodies assess the conformity of the software before being placed on the market by evaluating manufacturers’ technical documentation and quality management system with an increasing level of scrutiny based on the devices’ classification.^3,15^ In annex VIII of the MDR, rule 11 states that software intended to provide information that is used to take decisions with diagnostic or therapeutic purposes is classified as Class IIa, except if such decisions may cause a serious deterioration of a person’s health state or a surgical intervention (Class IIb) or when they can result in death or an irreversible deterioration of a person’s state of health (class III) (Figure, section B). Software that does not lead to decisions on diagnosis or therapy and that does not monitor physiological processes can be classified as a Class I device, allowing self-certification. An example of a class I software is an application calculating the user’s fertility status to predict ovulation. Moreover, the MDR takes on a product lifecycle-focused approach resulting in post-certification monitoring, annual surveillance audits of the manufacturers by notified bodies and re-certification procedures at least every five years.

## FDA vs MDR for CDS Functionalities

 Like the FDA, the MDR has leveraged the IMDRF risk-based framework for medical device software classification, but the MDR is more stringent: all software functionalities that classify as class I devices under FDA regulation are classified as at least class IIa devices under MDR regulation. The previous example of CDS for drug-drug interaction screening that is exempted from the Cures Act is at least classified as class IIa medical software in EU’s MDR. Since the application is used to guide the treatment of the patient, it will either belong to class IIa, IIb or III depending on how developers describe and notified bodies evaluate the intended use. Not detecting a drug-drug interaction may have no consequences but can also result in adverse drug events and worst case even in death. The highest potential risk will determine the risk profile. However, the risk-based categorization seems not fully defined, thereby creating opportunity for subjective interpretation.

 Like the US case, different European organizations raised awareness around the idea of regulating CDS applications too strongly.^16,17^ The FDA acknowledged such concerns with the draft guidance on CDS, but so far, the EU has not yet issued a specific statement concerning MDR and CDS risk classification. Most organizations think MDR will hamper software development, will limit their focus of producing software because of the administrative burden and will limit the access of these CDS applications to the market.^16,17^ The authors of this paper agree that it is a good idea to regulate medical software, but one should be aware of introducing a possible inequity between “large” and “small” companies and/or academic institutions that develop medical software in fulfilling the administrative part in terms of quality and safety. Both human resources and financial constraints may for example prevent hospitals from developing a homegrown healthcare information system with integrated medical device software functionalities (eg, CDS).

 The IMDRF is a consultative body between international stakeholders aiming to accelerate international medical device regulatory harmonization and convergence. IMDRF has the ability to play a leading role in the harmonization process of the USA’s and Europe’s viewpoint on CDS regulation. Countries and continents can aim to adopt similar definitions, but the implementation of regulatory frameworks remains the responsibility of the FDA for the Cures Act and EU government for the MDR legislation.

## Remaining Issues

 In our opinion neither American nor European authorities have clarified all issues concerning the regulation of CDS. The United States has gone through a process of revising the Cures Act, where over time risk categories were included and lower risk categories were assigned to medical software for which a user can understand or evaluate the basis of a clinical recommendation.

 In Europe, the MDR has not undergone similar major changes (yet). It is believed that EU’s regulation is larger in scope compared to the United States.^18^ Returning to the example of CDS for drug-drug interactions, the authors of this paper believe that the exemption in the United States and stringent regulation in Europe can lead to less CDS innovation in Europe compared to the United States. Currently, only 23 notified bodies spread over 11 different countries in the EU are accredited to regulate all applications for medical devices.^19^ Moreover, these notified bodies are responsible for evaluating both software applications and all other medical devices. Software vendors in countries without a notified body, are forced to use notified bodies located in another country and may not be able to apply in their own language. This also implies that the number of applications will be larger for these notified bodies, leading to a larger workload and potentially delayed market access to new technologies.

 When looking at the risk classification both in the United States and Europe, it is not clear how this classification is objectively assessed. In our opinion, medical software manufacturers will seek to certify their application in the lowest risk class possible. As mentioned earlier, this classification depends on the documentation describing the intended use and the risk evaluation procedure and on the evaluation of this documentation by the notified body.

 Furthermore, CDS may also include black-box solutions incorporating artificial intelligence. Do these solutions then automatically fall in the highest risk category, since the basis of that clinical decision is not directly understandable, or will the risk categorization depend more on the type of application where the artificial intelligence is used for? Or will the display of all data input elements be considered sufficient to independently review the basis of a recommendation? Will the MDR also take this approach? Uncertainty also remains on the threshold for recertification. Does an update of the algorithms require a recertification by a notified body, even if the intended use remains the same?

 These are questions that require answers before being able to apply for certification. A guidance document with an extensive list of applications and the corresponding risk category could make it easier and more comprehensible for (future) developers to understand the risk categorization. In the United States, the FDA already created such a list in which manufacturers and users of these devices can find to what risk class the device belongs according to the 21st Century Cures Act.^20^ However, the authors of this paper note that two medical software devices with the same intended use may differ substantially leading to different risk evaluations. For example, software for checking drug-drug interactions might be knowledge-based or non-knowledge based. The former category uses fixed IF-THEN rules that are programmed in the system to decide on an action (eg, alert), while the decisions of the latter category are based on more complex algorithms using for example, a statistical model (eg, logistic regression model) or a black-box machine learning algorithm where the basis of the decision is much more difficult to track. Both solutions have the same purpose but may have to evolve in a different risk evaluation in our opinion.

 Designing a regulatory framework that achieves the right balance between promoting innovation and fast market access on one side and ensuring safety and quality on the other side is very challenging. Both the United States and Europe responded to the initial need for a new regulatory framework. Future direction should go to providing sufficient guidance on how to fulfill a certification procedure from start to finish. Many consultancy companies are taking advantage of the complexity by offering assistance, but this places large costs on small market players. The goal of promoting innovation and fast market access might be bypassed. The authors understand that different risk evaluations will lead to different procedures, but manufacturers should at least find the appropriate information on where to start and what exactly needs to be done in order to meet the legislation requirements. Within the next few years, we will be able to evaluate the effect of these laws on the clinical translation of innovative CDS software systems.

## Ethical issues

 Not applicable.

## Competing interests

 Authors declare that they have no competing interests.

## Authors’ contributions

 All authors contributed to the theoretical concept of this viewpoint paper. SVL and KMM drafted the first version of the manuscript and all three authors critically reviewed the manuscript and gave their approval before submission.

## Funding

 KMM is supported by a grant of the Research Foundation Flanders (FWO) under grant number [1S39820N]. This funding body had no role in any part of the work.
